# Utilization of SARS-CoV-2 Wastewater Surveillance in Africa—A Rapid Review

**DOI:** 10.3390/ijerph19020969

**Published:** 2022-01-15

**Authors:** Tafadzwa Dzinamarira, Grant Murewanhema, Patrick Gad Iradukunda, Roda Madziva, Helena Herrera, Diego F. Cuadros, Nigel Tungwarara, Itai Chitungo, Godfrey Musuka

**Affiliations:** 1 School of Health Systems & Public Health, University of Pretoria, Pretoria 0002, South Africa; 2 ICAP at Columbia University, Harare, Zimbabwe; gm2660@cumc.columbia.edu; 3 Unit of Obstetrics and Gynaecology, Department of Primary Health Care Sciences, Faculty of Medicine and Health Sciences, University of Zimbabwe, Harare, Zimbabwe; gmurewanhema@yahoo.com; 4 London School of Hygiene and Tropical Medicine, University of London, London WC1E 7HU, UK; gadpatrickiradukunda@gmail.com; 5 School of Sociology and Social Policy, University of Nottingham, Nottingham NG7 2RD, UK; roda.madziva@nottingham.ac.uk; 6 School of Pharmacy and Biomedical Sciences, University of Portsmouth, Portsmouth PO1 2UP, UK; helena.herrera@port.ac.uk; 7 Department of Geography and Geographic Information Science, University of Cincinnati, Cincinnati, OH 45221, USA; cuadrodo@ucmail.uc.edu; 8 Department of Health Studies, University of South Africa, Pretoria 0002, South Africa; 34846751@mylife.unisa.ac.za; 9 Chemical Pathology Unit, Department of Laboratory Diagnostic and Investigative Sciences, Faculty of Medicine and Health Sciences, University of Zimbabwe, Harare, Zimbabwe; ichitungo@medsch.uz.ac.zw

**Keywords:** COVID-19, SARS-CoV-2, wastewater, surveillance, Africa

## Abstract

Wastewater-based epidemiology for SARS-CoV-2 RNA detection in wastewater is desirable for understanding COVID-19 in settings where financial resources and diagnostic facilities for mass individual testing are severely limited. We conducted a rapid review to map research evidence on the utilization of SARS-CoV-2 wastewater surveillance in Africa. We searched PubMed, Google Scholar, and the World Health Organization library databases for relevant reports, reviews, and primary observational studies. Eight studies met the inclusion criteria. Narrative synthesis of the findings from included primary studies revealed the testing methodologies utilized and that detected amount of SARS-CoV-2 viral RNA correlated with the number of new cases in the studied areas. The included reviews revealed the epidemiological significance and environmental risks of SARS-CoV-2 wastewater. Wastewater surveillance data at the community level can be leveraged for the rapid assessment of emerging threats and aid pandemic preparedness. Our rapid review revealed a glaring gap in the primary literature on SARS-CoV-2 wastewater surveillance on the continent, and accelerated and adequate investment into research is urgently needed to address this gap.

## 1. Introduction

Coronavirus disease 2019 (COVID-19) was first reported in Africa on 14 February 2020 [1]. While Africa’s response to the subsequent pandemic caused by this virus has been admirable [2,3,4,5], the continent continues to face challenges due to its limited resources. Specifically, the lack of local biotechnological production and limited research capacity or expertise in speciality fields has resulted in African countries being unable to conduct sufficient testing and focused research studies related to disease transmissibility relevant to the local context [6]. For this reason, given the importance of gathering data relevant to this region, there is a need to adopt and utilize sustained efficient and accessible COVID-19 epidemiological surveillance systems in Africa.

The epidemiological surveillance of pathogens in wastewater systems has significantly contributed to the surveillance of highly transmissible infectious diseases since its introduction in the 19th century. Wastewater surveillance for *Vibrio cholerae* [7], *Polio virus* [8], *Sallmonella tythi* [9], and many other pathogens has contributed to early warning systems (EWS) and allowed for informed and timely public-health responses [10].

Since the emergence of COVID-19, extensive research has been conducted to understand the viral structure of the causative agent, SARS-COV-2 [11], its survival in different external environments [12,13], and the clinical manifestations of COVID-19 [14,15,16]. Numerous studies have established the need for SARS-CoV-2 wastewater surveillance to better inform public health responses outside Africa [17,18,19,20]. SARS-CoV-2 was reported in solid waste, including faecal sludge from non-flushing on-site sanitation systems in Italy [17], sewage in the Netherlands [18], raw/untreated water in Australia [21], and drinking water supply in Mexico [20].

In general, wastewater surveillance of viral pathogens has several challenges. The complexity of wastewater matrices, the dilute nature of biomarkers in wastewater, difficulty in pinpointing the suitable sample locations, and the need for effective virus-concentrating methods often limit this method’s ability for quantitative predictions from the viral RNA [22,23]. Despite these challenges, findings from two reviews [24,25] that included studies up to July 2021 concluded that wastewater monitoring could be a useful tool for tracking COVID-19 spread.

While epidemiology for SARS-CoV-2 RNA detection in wastewater is attractive for understanding COVID-19 in settings where financial and diagnostic resources for testing are severely limited, there is a lack of research evidence on the utility of such techniques in Africa. The availability of reliable data on COVID-19 burden and transmission is critical for prioritising and deploying scarce resources, including personal protective equipment (PPE) and emergence response systems [26]. Thus, this study aimed to map research evidence on SARS-CoV-2 wastewater surveillance in Africa to inform future research and synthesize gaps and opportunities.

## 2. Methodology

### 2.1. Information Sources and Literature Search

We searched PubMed, Google Scholar, and the World Health Organization library databases for relevant studies. The key search terms included “Coronavirus”, “Covid-19”, “2019-nCoV,” “SARS-CoV-2”, “wastewater”, “Africa”, “epidemiology”, and “surveillance”. All database searches were conducted on 5 December 2021. Due to the rapid nature of this review, a modified population, intervention, control, and outcomes (PICO) framework informed the development of the search strategy to ensure that the boundaries of the research question were clearly defined:

Search # 1—population (studies conducted in African countries),

Search # 2—intervention ~ the environmental matrix of interest (wastewater-based surveillance, role in management/monitoring of the COVID-19 pandemic),

Search # 3—outcomes (the utilization of, acceptability of, and significance of wastewater-based surveillance in the management/monitoring of the COVID-19 pandemic).

The full search strategy with Medical Subject Headings (MeSH) descriptors and truncation is presented in Table 1. The reference lists of all full-text articles screened were searched for relevant studies.

### 2.2. Study Selection and Inclusion Criteria

We searched for reports, reviews, and primary observational studies (case–control, case–cross-over, cross-sectional, and cohort). The review included studies conducted within Africa that described the utilization of SARS-CoV-2 wastewater surveillance.

### 2.3. Screening Process

We developed a screening criterion a priori for each of the three stages: title, abstract, and full text. Two researchers screened the articles independently. Differences in screen results at the full-text stage were resolved by discussion. Covidence, an online tool for conducting various types of reviews (www.covidence.org), was used to review the titles and abstracts for inclusion/exclusion based on the criteria described in the modified PICO framework. Next, articles were single screened during full text using the same inclusion/exclusion criteria.

### 2.4. Data Abstraction and Synthesis

A data abstraction form was developed, discussed, and revised a priori. Data extraction was completed using the following endpoints: (1) country; (2) study aim; (3) study design (if applicable); (4) utility/description of SARS-CoV-2 wastewater surveillance methods; (5) any other significant findings. Only one reviewer abstracted data from the included studies. For collating, summarizing, and reporting the findings, first, the reviewers familiarized themselves with the content of the articles. Second, findings reported in the papers were grouped into categories based on the reported findings, and a narrative was provided.

## 3. Results

**Figure 1:**
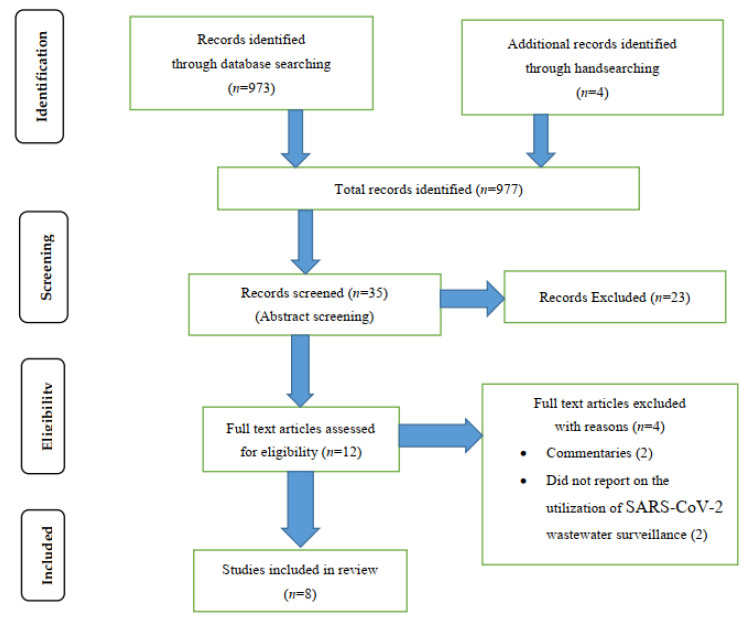
PRISMA flow diagram.

Our initial keyword database search found 977 potentially eligible articles (39 from PubMed, 918 from Google Scholar, 16 from the WHO databases, and 4 from handsearching). Following title screening, 44 articles were eligible for inclusion in abstract screening. These articles were imported into Convidence and nine duplicates were removed, leaving 35 articles included in the abstract screening. A total of 23 studies were excluded following abstract screening, leaving 12 articles [27,28,29,30,31,32,33,34,35,36,37,38] for full-text screening. Four articles [27,28,30,34] were excluded after full-article screening, leaving eight articles for data extraction (Supplementary Table S1). Of the excluded articles, two were commentaries [30,34], and one did not report on the utilization of SARS-CoV-2 wastewater surveillance [27,28]. More details are presented in the Preferred Reporting Items for Systematic Reviews and Meta-Analyses (PRISMA) flowchart in Figure 1.

### 3.1. Characteristics of Included Studies

Of the eight articles included in the study, four were from South Africa [29,31,33,38], two from Morocco [35,36], and one each from Cameroon [32] and Tunisia [37]. Three were cross-sectional studies [29,33,37], four were reviews [32,35,36,38], and one was a short communication/proof-of-concept piece [31]. More details are presented in Supplementary Table S1.

### 3.2. Study Findings

#### 3.2.1. Testing Methodologies and Target Genes

Of the four studies that collected and analyzed wastewater, three were conducted in South Africa [29,31,33] and one in Tunisia [37]. In all four studies, SARS-CoV-2 was extracted from municipal wastewater. A study conducted to evaluate the temporospatial migration and attenuation of the SARS-CoV-2 virus using the polymerase chain reaction (PCR) in municipal wastewater in South Africa [29] utilized the 2019-nCoV genesig^®^ Advanced Kit (Primerdesign Ltd., Plymouth Meeting, PA, USA). Two other studies conducted in South Africa [31,33] utilized the RT-PCR (Whitehead Scientific, Integrated DNA Technologies, AI, USA) extraction methodology. In Tunisia, Jmii et al. [37] used the Allplex 2019-nCoV kit (Seegene, Seoul, Korea) and the QuantiTect virus Kit (Qiagen, Hilden, Germany) for extraction. Of the three cross-sectional studies, two targeted the RNA-dependent RNA polymerase (RdRp) gene [29,37].

#### 3.2.2. Detected Amount of SARS-CoV-2 Viral RNA Correlated with Number of New Cases in the Studied Areas

In two studies [33,37], SARS-CoV-2 RNA was detected in raw and treated wastewater collected from different municipalities. In both studies, SARS-CoV-2 viral RNA correlated with the number of new cases in the studied areas. In Street et al.’s study in South Africa [33], there was an overall decrease in the amount of detected viral RNA over the study period, associated with a declining number of newly identified COVID-19 cases. In Tunisia, an increase in the amount of detected viral RNA over the study period was associated with a rapid rise in the number of newly identified COVID-19 cases in the studied area [37]. These findings supported wastewater surveillance as an EWS towards COVID-19 infection in the communities.

#### 3.2.3. SARS-CoV-2 in Wastewater: Epidemiological Significance and Environmental Risks

Four studies explored the epidemiological significance of SARS-CoV-2 in wastewater and the associated environmental risks [32,35,36,38]. Although not backed by study findings, one study suggested that SARS-CoV-2 wastewater surveillance could be a cost-effective, rapid, and reliable source of information on the spread of SARS-CoV-2 and its variants in the population [32]. Two studies revealed that wastewater surveillance could enhance genomic and epidemiologic surveillance with independent and complementary data to inform public health decision-making during ongoing pandemics [32,38].

Two studies [35,36] revealed how the occurrence of SARS-CoV-2 in the environment poses public health risks at sites of sewage products disposal and reuse, especially in low-income countries with inadequate sanitation, where direct discharge and reuse of raw sewage are standard practices. This is in the realization that faecal–oral transmission is also considered as a potential route for SARS-CoV-2 transmission.

## 4. Discussion

Wastewater surveillance has been identified as a potential leading indicator of changes in the COVID-19 prevalence in communities [39]. The major strength of wastewater surveillance is its independence to healthcare provision of clinical testing. This can be very informative in some African settings, where access to clinical care services is limited due to insufficient capacity alongside lack of medical insurance for the majority. Data from wastewater surveillance, which indicates community infection trends, can be used to allocate or prioritise clinical testing resources efficiently, investigate caveats in traditional surveillance, formulate targeted risk-communication messaging, and forecast emerging clinical resource requirements [40]. Sub-Saharan Africa, where clinical PCR testing resources are inadequate [41], could substantially benefit from robust wastewater surveillance systems. Wastewater surveillance data at the community level can be leveraged for the rapid assessment of emerging threats and aid pandemic preparedness.

Regarding testing methodologies, SARS-CoV-2 genetic material was extracted from municipal water in all the four studies that researched this, three of which were based in South Africa. The major difference between South Africa and other African countries is its well-developed sewage and reticulation system, with access to modern sanitation methods for significant proportions of the population that facilitated studies of wastewater genetic material. In other countries, such as Zimbabwe, where the majority of the population is rural with no access to modern sanitation methods, access to wastewater surveillance would be mainly limited to urban settings with modern sanitation systems. Whilst useful urban community SARS-CoV-2 infection trends could be obtained in these countries, significant proportions of communities would be underrepresented, with a resultant reduction in wastewater surveillance data in these settings. However, while it is not possible to survey those places where there are no water plants, surveying major cities might facilitate the detection of trends in virus spread that would also be relevant to rural populations. Furthermore, even with the limited data that can be obtained from settings without modern facilities, it would be possible to successfully supplement other data to enable a prompter response and targeted deployment of resources in these areas. In the future, as infrastructure is improved in the African continent, wastewater surveillance will become more informative, not just for SARS-CoV-2 community infections trends but for other epidemic-prone diseases such as typhoid and cholera.

Our findings revealed that the 2019-nCoV genesig, Advanced Kit, RT-PCR qRT-PCR, Allplex 2019 nCOV kit, and the QuantiTect virus kit were used across the different studies to extract SARS-CoV-2 genetic material in wastewater in African countries. The RNA-dependent RNA polymerase (RdRp) gene was targeted for extraction in two of the three cross-sectional studies. Part of the reason there is a paucity of studies across the African continent is a lack of appropriate testing technologies and limited resources [6]. African countries have struggled with testing that involves nucleic acid amplification, especially in the public health sector [6]. Besides conventional RT-PCR, countries also had to utilize GeneXpert technologies to improve the testing capacity [42]. Unfortunately, a global shortage of cartridges has resulted in the underutilization of these pre-existing GeneXpert technologies for tuberculosis testing. For clinical testing of COVID-19 patients and surveillance, African countries have migrated to cheaper rapid antigen-detection test kits [43], which have not been utilized in wastewater surveillance. There is a need for African countries to make the best use of limited resources by identifying where these may be needed via SARS-CoV-2 wastewater surveillance, which does not rely on external indicators, only on relatively cheap testing of wastewater.

Our review confirmed that the detected amount of SARS-CoV-2 RNA correlated positively with an increased incidence COVID-19 cases at the community level. This finding agrees with other studies conducted elsewhere [39,40]. This provides direct evidence that frequent wastewater surveillance can be helpful as an early warning system to trigger community-level surveillance and responses to imminent COVID-19 resurgences. However, the cross-sectional nature of the included studies limits a definitive conclusion, and prospective studies would be more helpful in determining the utility of wastewater surveillance as an early warning system. This is coupled with the lack of representative studies, as three studies are insufficient to determine the excretion patterns of SARS-CoV-2 genetic material across a continent with heterogeneous populations.

Of the studies that explored the epidemiological significance of SARS-CoV-2 in wastewater and the associated environmental risks, one established wastewater surveillance as cost-effective, rapid, and reliable with regards to providing SARS-CoV-2 community infection trends and the possible detection of newer variants [32]. It has been argued that genomic sequencing should be an integral component of COVID-19 surveillance to inform the emergence of variants of interest and variants of concern early enough and devise appropriate public health policy and strategies to mitigate against their widespread transmission, given their potential to evade preventative measures such as vaccination [44]. Wastewater surveillance might enable timely detection of emerging variants of SARS-CoV-2. In that case, it becomes an indispensable tool for surveillance, but in resource-limited settings in Africa, this would need to be accompanied by increasing the genomic sequencing capacity [44,45]. Two of the studies also showed that SARS-CoV-2 wastewater surveillance is a useful complement to genomic and epidemiologic surveillance [32,38], implying that it cannot stand alone as a surveillance tool, but when utilized together with the other aspects of surveillance, provides useful information to effectively inform public health. It is congruent with the conclusions of the World Health Organization [46].

The availability of only eight eligible studies based in the African continent, from only four countries (South Africa, Morocco, Cameroon, and Tunisia) highlight the lack of adequate exploration of this vital aspect of surveillance in the continent. To better inform public health policy and strategy on the continent, more studies from a variety of countries are required to generate representative data and valid conclusions. Only three of the eight studies retrieved were primary studies, of a cross-sectional design, with 50% being reviews. While there is substantial evidence of primary studies being conducted elsewhere [17,18,19,20,21,39,40], there is clearly a glaring gap of primary literature on SARS-CoV-2 wastewater surveillance on the continent, and accelerated and adequate investment into research is urgently needed to address this gap. It is also worth noting that half of the included studies were conducted in South Africa. South Africa is a middle-income country and is better placed ahead of its many other African counterparts in providing clinical testing services. However, the cost of PCR has generally been high in the country [47].

Two studies [35,36] revealed the environmental risks associated with the occurrence of SARS-CoV-2 in the environment. The excretion of viable SARS-CoV-2 virions could pose a serious severe infection hazard in communities where sewage systems are not efficient and raw sewage spills into communities. There is evidence, albeit limited, of the possibility of faecal–oral SARS-CoV-2 transmission [48], and two of the studies in this review alluded to the possibility of this risk. This calls for responsible municipalities to ensure efficient sewage systems with minimal spillage of raw contents into communities, where children play in the streets. In less affluent countries in Africa, vending is also rife in the streets. This would pose a risk of precipitating community transmission, and that is why ensuring adequate water, and sanitation systems, is a significant public health priority.

This rapid review has some limitations worth noting. Due to the rapid nature of this review, only three databases were searched. However, multiple search terms were utilized and retrieved relevant articles in PubMed. The rapid review provides essential insights into the lack of robust studies on wastewater surveillance in Africa. Properly designed longitudinal studies with an adequate follow-up period would provide more conclusive evidence of the utilization of SARS-CoV-2 wastewater surveillance in Africa. The lack of sufficient research funding and testing capacity needs to be addressed to allow more studies to be conducted. The studies retrieved in this review lacked representativeness, as they represented only four African countries out of the whole African continent. The socioeconomic and population characteristics may differ significantly across these countries, limiting the external validity and generalizability of the findings.

## 5. Conclusions

Wastewater surveillance data at the community level can be leveraged for the rapid assessment of emerging threats and aid pandemic preparedness. Our rapid review revealed a glaring gap in the primary literature on SARS-CoV-2 wastewater surveillance on the continent and accelerated and adequate investment into research is urgently needed to address this gap.

## Figures and Tables

**Table 1:** Search strategy with articles retrieved from PubMed electronic database.

Scheme	Query	PubMed (5 December 2021)
#1	**African countries filter:** ((((Angola OR Benin OR Botswana OR “Burkina Faso” OR Burundi OR Cameroon OR “Cape Verde” OR “Central African Republic” OR Chad OR Comoros OR Congo OR “Democratic Republic of Congo” OR Djibouti OR “Equatorial Guinea” OR Eritrea OR Ethiopia OR Gabon OR Gambia OR Ghana OR Guinea OR “Guinea Bissau” OR “Ivory Coast” OR “Cote d’Ivoire” OR Kenya OR Lesotho OR Liberia OR Madagascar OR Malawi OR Mali OR Mauritania OR Mauritius OR Mozambique OR Namibia OR Niger OR Nigeria OR Principe OR Reunion OR Rwanda OR “Sao Tome” OR Senegal OR Seychelles OR “Sierra Leone” OR Somalia OR “South Africa” OR Sudan OR Swaziland OR Tanzania OR Togo OR Uganda OR “Western Sahara” OR Zambia OR Zimbabwe OR “Central Africa” OR “Central African” OR “West Africa” OR “West African” OR “Western Africa” OR “Western African” OR “East Africa” OR “East African” OR “Eastern Africa” OR “Eastern African” OR “South African” OR “Southern Africa” OR “Southern African” OR “sub Saharan Africa” OR “sub Saharan African” OR “sub-Saharan Africa” OR “sub-Saharan African”	475,901
#2	**Intervention or the environmental matrix of interest filter:** “Waste Water” OR Sewage OR Wastewater OR “wastewater treatment plant” OR WBE OR “environmental surveillance network” OR “Environmental surveillance system” OR “Wastewater surveillance” OR “Wastewater-based epidemiology” OR “Droplet digital PCR” OR sewage	129,082
#3	**Outcomes intervention filter:** “Human Coronavirus” OR “Severe acute respiratory syndrome” OR “SARS Virus” OR “COVID-19” OR “COVID19” OR HCoV OR 2019-nCoV OR SARS-CoV OR SARS-CoV-2 OR “severe acute respiratory syndrome coronavirus 2” OR nCoV OR “2019 novel coronavirus” OR “novel coronavirus” OR “coronavirus 2019” OR “Novel coronavirus 2019” OR “Wuhan coronavirus” OR “novel coronavirus disease”	209,566
**#4**	#1 AND #2 AND #3	39

## Data Availability

All data related to this study are presented in the manuscript and supplementary files.

## Supplementary Materials

The following are available online at https://www.mdpi.com/article/10.3390/ijerph19020969/s1, Table S1: Characteristics of included studies.
